# Case Report: A Novel Gross Deletion in *PAX3* (10.26 kb) Identified in a Chinese Family With Waardenburg Syndrome by Third-Generation Sequencing

**DOI:** 10.3389/fgene.2021.705973

**Published:** 2021-08-11

**Authors:** Jie-Yuan Jin, Lei Zeng, Bing-Bing Guo, Yi Dong, Ju-Yu Tang, Rong Xiang

**Affiliations:** ^1^Department of Orthopaedics, Xiangya Hospital of Central South University, Changsha, China; ^2^School of Life Sciences, Central South University, Changsha, China; ^3^Hunan Key Laboratory of Animal Models for Human Diseases, School of Life Sciences, Central South University, Changsha, China; ^4^Hunan Key Laboratory of Medical Genetics, School of Life Sciences, Central South University, Changsha, China

**Keywords:** *PAX3*, Waardenburg syndrome, third generation sequencing, structural variants, deletion

## Abstract

Waardenburg syndrome (WS) is a group of autosomal-dominant hereditary conditions with a global incidence of 1/42,000. WS can be categorized into at least four types: WS1–4, and these are characterized by heterochromia iridis, white forelock, prominent nasal root, dystopia canthorum, hypertrichosis of the medial part of the eyebrows, and deaf-mutism. WS3 is extremely rare, with a unique phenotype (upper limb abnormality). Heterozygous mutations of *PAX3* are commonly associated with WS1, whereas partial or total deletions of *PAX3* are often observed in WS3 cases. Deletions, together with insertions, translocations, inversions, mobile elements, tandem duplications, and complexes, constitute structural variants (SVs), which can be fully and accurately detected by third-generation sequencing (TGS), a new generation of high-throughput DNA sequencing technology. In this study, after failing to identify the causative gene by Sanger sequencing, SNP-array, and whole-exome sequencing (WES), we finally detected a heterozygous gross deletion of *PAX3* (10.26kb, chr2: 223153899-223164405) in a WS family by TGS. Our description would enrich the genetic map of WS and help us to further understand this disease. Our findings also demonstrated the value of TGS in clinical genetics researches.

## Introduction

Waardenburg syndrome (WS) is a group of genetic conditions that mainly follow an autosomal dominant pattern, with a global incidence of 1/42,000 (Apaydin et al., 2004). WS can be phenotypically and genotypically categorized into at least four types: WS type I–IV. WS type I (WS1) and WS type II (WS2) are the most common types, whereas WS type III (WS3) has rarely been reported (Ahmed Jan et al., 2021). The prominent characteristics include heterochromia iridis, white forelock, prominent nasal root, dystopia canthorum, hypertrichosis of the medial part of the eyebrows, and deaf-mutism (Gowda et al., 2020). Except for common features, patients with WS3 have upper limb abnormalities, such as finger contractures, cutaneous syndactyly, and hypoplasia of the bones of the upper limbs and wrists (Ahmed Jan et al., 2021).

WS is caused by the lack of melanophores in the eyes, skin, cochlea, and hair, which in itself originates from mutations in different genes affecting neural crest function (Alehabib et al., 2020). The known causative genes of WS include *PAX3, MITF, WS2B, WS2C, SNAI2, EDNRB, EDN3, SOX10*, and *TYR*; among them, *PAX3* is the most classic, and its mutations or gross deletions produce WS1 or WS3 (Yu et al., 2020). To date, heterozygous mutations of *PAX3* are commonly associated with WS1, whereas partial or total deletions of *PAX3* are often observed in WS3 cases (Ahmed Jan et al., 2021).

Deletions, together with insertions, translocations, inversions, mobile elements, tandem duplications, and complexes, constitute structural variants (SVs; defined as DNA variants ≥50 bp) (Alkan et al., 2011). Similar to single nucleotide variations and small insertions/deletions, SVs are highly polymorphic and widely distributed in the genome, which may cause genetic diseases and tumors, such as thalassemia, red-green color blindness, and WS (Stankiewicz and Lupski, 2010; Genomes Project et al., 2015). Although SVs are important, many challenges exist in their detection because of the various types and sizes. Traditional detection methods, such as multiplex ligation-dependent probe amplification (MLPA), microarray, and whole-exome sequencing (WES), cannot fully and accurately uncover SVs. Third-generation sequencing (TGS) is a new type of DNA high-throughput sequencing, based on single-molecular real-time sequencing technology, to identify SVs (Xiao and Zhou, 2020).

In this study, we reported a Chinese family of three patients with WS1/3. After receiving a series of negative results from Sanger sequencing, SNP-array, and WES, we finally detected a heterozygous gross deletion of *PAX3* (10.26kb, chr2: 223153899-223164405) in patients using TGS. To the best of our knowledge, this deletion has not been reported, and it enriches the genetic map of WS and helps us to further understand this disease. This research also demonstrated the value of TGS in clinical genetics researches.

## Materials and Methods

### Patients and Subjects

This research was approved by the Review Board of Xiangya Hospital of Central South University. Written informed consents were obtained from the patients and their guardians, and all subjects consented to participation in this study and to image publication. Blood samples were collected from the proband and the blood relations.

### DNA Extraction

Genomic DNA was extracted from the peripheral blood samples using the DNeasy Blood & Tissue Kit (Qiagen, Valencia, CA, USA).

### Polymerase Chain Reaction and Sanger Sequencing

*PAX3* reference sequences and coding regions (NM_181457.4) were obtained from NCBI (https://www.ncbi.nlm.nih.gov/gene/5077), and primers were designed using IDT (http://sg.idtdna.com/Primerquest/Home/Index) (Supplementary Table 1). Target sequences were amplified using polymerase chain reaction (PCR) and detected.

### Agarose Gel Electrophoresis

PCR products were electrophoresed on a 1% agarose gel. Gels were stained with goldview and photographed with a standard UV-transilluminator, SageCreation SmartChemi.

### SNP-Array Analysis

SNP-array analysis was performed according to the methods described by Jin et al. (2019), with slight modifications where necessary (Jin et al., 2019).

### Whole-Exome Sequencing

Berry Genomics Company Limited (Chengdu, China) performed exome capture, high-throughput sequencing, and common filtering, as described in a previous article (Jin et al., 2020). Co-segregation analysis was conducted in the family, and candidate genes were further screened using OMIM (https://www.ncbi.nlm.nih.gov/omim/) and HGMD (http://www.hgmd.cf.ac.uk/ac/search.php).

### Third-Generation Sequencing

Detailed instructions of TGS are provided in the Supplementary Materials, “Protocol: Third-generation sequencing.” Berry Genomics Company Limited (Chengdu, China) provided the TGS service. The SMRT bell library was constructed and sequenced using PacBio SMRT Technology (Xiao and Zhou, 2020). Subreads were obtained by deleting short polymerase reads and adapter sequences and were then compared with hg19 using the Smrtlink8.0 Pbsv process, and SV data were acquired using SV detection software. SV results were annotated using the Berry Enliven® system, OMIM, and DECIPHER (https://decipher.sanger.ac.uk/index).

## Results

### Clinical Features

**Figure 1:**
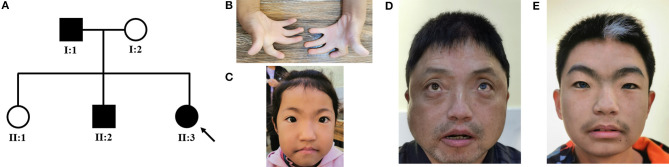
**(A)** Pedigree of the WS family with three patients. The black symbols represent the affected members, and the arrow indicates the proband. **(B–E)** Partial phenotypes of three patients. Proband (II:3) had bilateral finger contractures with mild cutaneous syndactyly **(B)**, dystopia canthorum, and faint synophrys **(C)**; her father (I:1) had dystopia canthorum, bright blue eyes, and prominent nasal root **(D)**; her brother (II:2) had dystopia canthorum, bilateral bright blue eyes, synophrys, white forelock, and prominent nasal root **(E)**.

We identified a Chinese family with WS (Figure 1A). The proband (II:3) was a 9-year-old girl, who was admitted to our hospital for bilateral finger contractures (Figure 1B). Besides flexion contractures of 10 fingers, she had mild cutaneous syndactyly. We observed that the girl had dystopia canthorum and faint synophrys (Figure 1C). Tracing back her family history, we found that her mother and sister were unaffected, while her father (I:1) and brother (II:2) had similar facial features, including dystopia canthorum, bright blue eyes (I:1's left eye, and bilateral eyes in II:2), synophrys (I:1 shaping eyebrows), white forelock (I:1 dying hair), and a broad nasal root (Figures 1D,E). Furthermore, I:1 had arthrogryposis of the bilateral fifth fingers, and II:2 was diagnosed with sensorineural deafness. The family showed no other genetic or infectious diseases. Hence, we suspected WS1 and WS3 in the family.

### Genetic Tests

**Figure 2:**
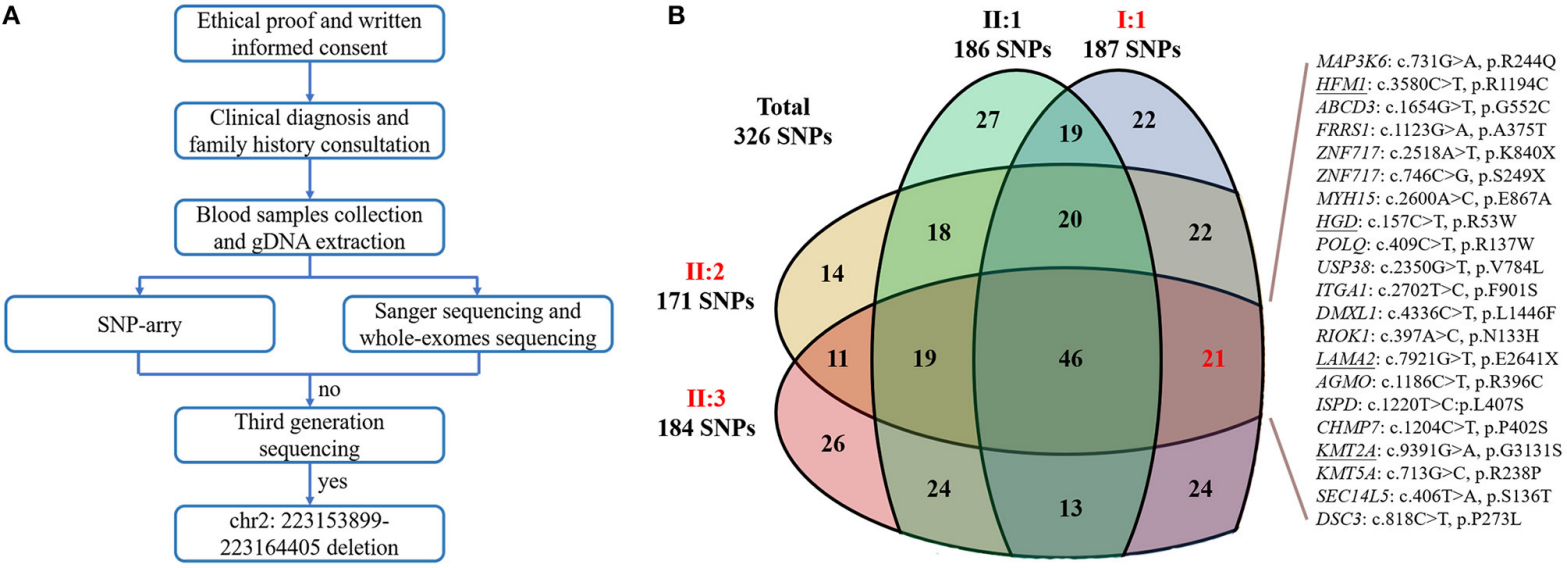
**(A)** The flow diagram of the present research. “no” indicates that we did not detect the causative gene; “yes” indicates that we identified the genetic defect. **(B)** The Venn diagram of whole-exome sequencing results. Red words represent the affected members; “SNPs” is an abbreviation of single nucleotide polymorphisms; underlines indicate the variants have been reported.

**Figure 3:**
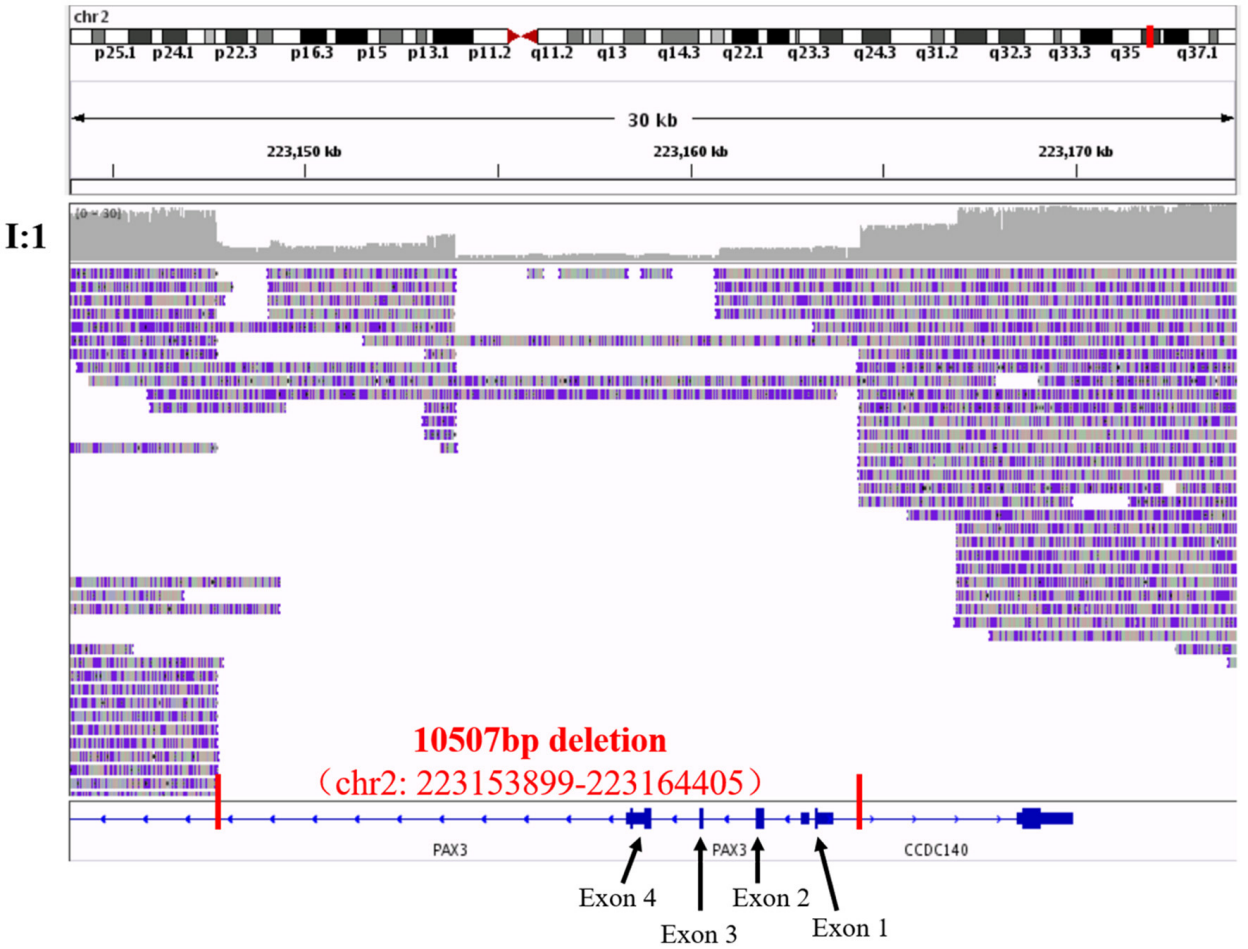
The visualization of third-generation sequencing result of Chr2: 223143899-223174405 in I:1 by IGV software. Red vertical lines cut out the region of the structural variant, and red words exhibited the details of this structural variant. Black arrows and words point out the exons of *PAX3* in the region.

**Figure 4:**
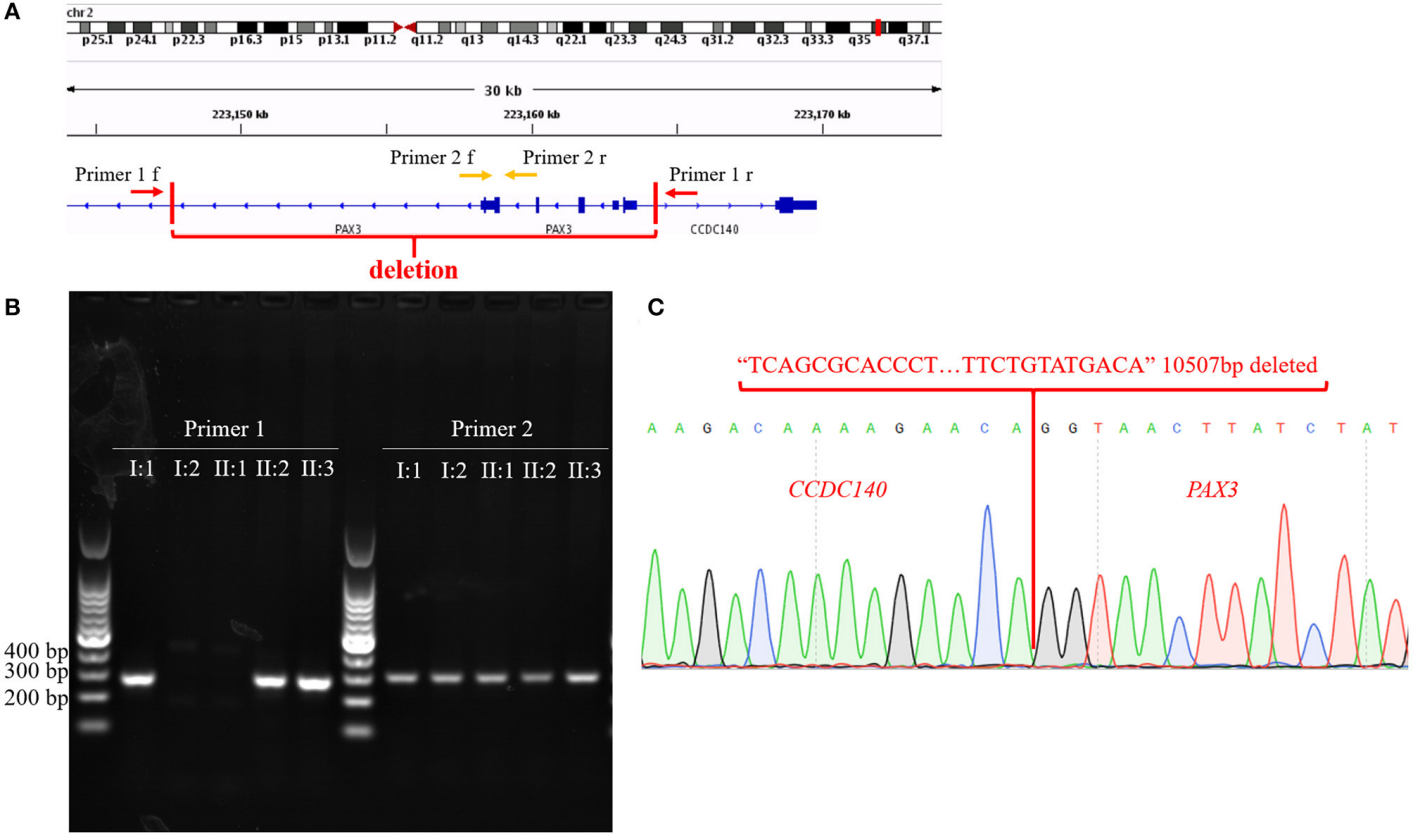
**(A)** The diagram of primer 1/2 pairs design. Arrows represent primer pairs; red vertical lines cut out the region of the structural variant. **(B)** Agarose gel electrophoresis results. **(C)** Sequencing results of sequences amplified according to Primer 1. Red sequence represents deleted sequence; red words indicate genes that correspond to the sequences.

To confirm our primary diagnosis, we implemented a genetic test program, as shown in Figure 2A, to identify the disease-causing gene of this family and assist in clinical diagnosis. Given that *PAX3* was the only known gene responsible for WS3, we applied Sanger sequencing to detect *PAX3* coding regions, but did not find any mutations in the proband. The SNP-array results from I:1 showed unfavorable copy number variation (CNV) data (Supplementary Table 2). Furthermore, the causative gene could not be identified using WES. Twenty-one variants in 20 genes were segregated from 326 SNPs, but none seemed a suspicious pathogenic mutation (Figure 2B). We observed that the coverage of exons 1–4 of *PAX3* in patients (I:1, II:2, and II:3) was conspicuously lower than that in normal subject (II:1) (Supplementary Figure 1). While this finding suggests a deletion in the exon region, the SNP array did not identify it. Thereafter, we used TGS to ascertain the pathogenesis of this family. A total of 51,035 SVs were identified in I:1 by TGS, including 6,768 translocations, 252 CNVs, 42 inversions, 21,007 deletions, 16,933 insertions, and 6,033 duplications (Supplementary Figure 2). Following filtration by OMIM and DECIPHER, we detected a heterozygous gross deletion of *PAX3* (10.26kb, chr2: 223153899-223164405) in I:1 (Figure 3). This region overlapped *PAX3* and *CCDC140*, including the promoter and exons 1–4 of *PAX3*. Agarose gel electrophoresis showed that the deletion co-segregated with the affected individuals, and this deletion was verified by Sanger sequencing (Figure 4). Hence, we reasoned that the *PAX3* deletion was the genetic lesion of this family, causing WS3 or WS1.

## Discussion

WS is an orphan genetic disease; WS3 is the rarest type (Ahmed Jan et al., 2021). In this study, we reported a WS family with obvious heterogeneity. The father (I:1) and brother (II:2) had classical WS facial features, while craniofacial dysmorphism of the proband (II:3) was atypical. I:1 and II:3 had acral dysplasia without deafness, and the phenotype of the former was milder. No evident limb abnormality in II:2, but he was affected by deaf-mutism. The complexity of the symptoms seriously disturbed the clinical diagnosis. The family was diagnosed with WS1/3, mainly based on limb malformation and molecular diagnosis. The four types of WS exhibit common phenotypes and differences. Compared with WS1, the inner canthi of both eyes in patients with WS2 were normal, those with WS3 exhibit upper limb abnormalities, and those with WS type IV (WS4) had Hirschsprung disease (Wang et al., 2017; Ahmed Jan et al., 2021). Symptoms in I:1 and II:3 were more consistent with those in WS3, whereas II:2 was diagnosed with WS1.

Both WS1 and WS3 are caused by PAX3 defects. Heterozygous *PAX3* mutations have been identified in 80% of WS1 cases, whereas gross deletions in *PAX3* are often observed in WS3 (Boudjadi et al., 2018). A minority of *PAX3* mutations (either heterozygotes or homozygotes) can also cause WS3. For example, a heterozygous mutation, c.388_400del, was detected in patients with WS3 (Tekin et al., 2001). Zlotogora et al. (1995) and Wollnik et al. (2003) also reported cases where children with the homozygote in *PAX3* were born with WS3 in consanguineous WS1 families (Zlotogora et al., 1995; Wollnik et al., 2003). Correspondingly, gross deletions could also trigger WS1. Matsunaga et al. (2013) described a Japanese family with a 1,759–2,554 kb deletion including a whole *PAX3* gene, diagnosed with WS1 (Matsunaga et al., 2013). In this study, patients all harbored the 10.26 kb heterozygous deletion (10.26 kb, chr2: 223153899-223164405); we referred to this important evidence in our diagnosis. Patients from this family with either WS1 or WS3 triggered by the same genetic etiology indicated that a close relationship existed between these two diseases. In fact, WS3 can be regarded as an extreme presentation of WS1.

The gross deletion identified in this family spanned *PAX3* and *CCDC140*, including the promoter and exons 1–4 of *PAX3*. Two regulatory sequences occur 1.6 kb upstream of the transcription start site and intron 4, respectively, which were predicted to be affected (Boudjadi et al., 2018). We hypothesized that this SV would cause the non-transcription of the defective *PAX3*, similar to the haplotype, and further studies are required to verify this.

*PAX3* encodes the paired box gene 3, a member of the paired box family, which acts as a transcription factor or transcriptional inhibitory factor (Buckingham and Relaix, 2015). PAX3 is initially expressed in the neural tube, and subsequently confined to proliferative cells in the inner ventricular zone, which may significantly affect central nervous system development (Goulding et al., 1991). Therefore, some patients with WS1/3 exhibit moderate dysgnosia. Our patients exhibited normal intelligence. PAX3 contributes to embryonic myogenesis by providing a pool of myogenic progenitors. Satellite cells expressing myogenic progenitor–derived PAX3 are frequent in the forelimb, which may explain why WS3 cases, such as I:1 and II:3, had upper limb, but not lower limb, abnormalities (Buckingham and Relaix, 2015). Melanoblasts with PAX3 expression originate from neural crest cells present in the skin, hair follicles, and the developing multiple inner ear components, whose deficiency is a response to white forelock and deafness (Boudjadi et al., 2018). The varying phenotypic severity exhibited by our patients may have resulted from differences in genetic background and environmental factors.

The spectrum of human genetic variation ranges from a single base pair to large chromosomal events, and the rate of SVs is more frequent than that of point mutations (at least 1000–10000-fold). SV-triggered conditions have been referred to as genomic disorders, and a major mechanism of SV- conveying phenotypes is gene dosage, such as in the present WS family that was caused by the haploinsufficiency of PAX3 (Stankiewicz and Lupski, 2010). Inchoate SV testing technologies, such as karyotype analysis, fluorescence *in situ* hybridization (FISH), and MLPA, are low-resolution and low-throughput (Xiao and Zhou, 2020). Microarrays are limited in their ability to detect small dose-imbalance SVs on known genomes (Stosic et al., 2018). The SV detectability of next-generation sequencing (NGS) is very restricted because of the short reads and sequencing preferences of NGS. Alternately, TGS with advantages, such as extra-long reads (80 k) and no requirement for PCR, have been used to fully and accurately detect various types of SVs in whole genomes, while TGS is time-consuming for annotation and analysis, and is expensive (Xiao and Zhou, 2020). In this study, Sanger sequencing and SNP array did not detect *PAX3* deletion, and coverage analysis of WES suggested that an SV occurred in *PAX3*, but without exact data. Finally, we determined the 10.26 kb deletion using TGS, again demonstrating the value of TGS in clinical genetics research.

## Conclusion

In summary, we identified a novel gross deletion in *PAX3* (10.26 kb, chr2: 223153899-223164405) in a Chinese family with both WS1 and WS3 by TGS. Our description enriched the genetic map of WS, contributed to further understanding of this disease, and showed the significance of TGS in molecular diagnosis.

## Data Availability Statement

The original contributions presented in the study are included in the article/Supplementary Material, further inquiries can be directed to the corresponding author/s.

## Ethics Statement

The studies involving human participants were reviewed and approved by the Review Board of Xiangya Hospital of the Central South University. Written informed consent to participate in this study was provided by the participants' legal guardian/next of kin. Written informed consent was obtained from the individual(s) and minor(s)' legal guardian/next of kin, for the publication of any potentially identifiable images or data included in this article.

## Author Contributions

J-YJ analyzed the sequencing results. LZ diagnosed and collected samples and clinical information. B-BG extracted gDNA and performed PCR and agarose gel electrophoresis. YD designed primer pairs and analyzed the WES data. J-YT diagnosed, designed the experiment, and edited the draft. RX designed the experiment, curated data, and wrote the original draft. All authors contributed to the article and approved the submitted version.

## Conflict of Interest

The authors declare that the research was conducted in the absence of any commercial or financial relationships that could be construed as a potential conflict of interest.

## Publisher's Note

All claims expressed in this article are solely those of the authors and do not necessarily represent those of their affiliated organizations, or those of the publisher, the editors and the reviewers. Any product that may be evaluated in this article, or claim that may be made by its manufacturer, is not guaranteed or endorsed by the publisher.
