# Supplementary material for: Case Report: A Novel Gross Deletion in PAX3 (10.26 kb) Identified in a Chinese Family With Waardenburg Syndrome by Third-Generation Sequencing
Source: Front Genet. 2021 Aug 11;12:705973. doi: 10.3389/fgene.2021.705973 (PMC8385755; doi:10.3389/fgene.2021.705973)
Supplement: Supplementary file 1 [file Data_Sheet_1.PDF]

PACBIO®

Procedure & Checklist - Preparing gDNA Libraries Using the SMRTbell® Express Template Preparation Kit 2.0

This document provides an optimized procedure for preparing gDNA libraries with an average insert size of 10 kb or larger using the SMRTbell Express Template Prep Kit 2.0. This document has four main sections:

1. Recommendations for gDNA QC and quantification
2. Recommendations for shearing gDNA to the desired insert size
3. Enzymatic steps in preparation of the SMRTbell library preparation
4. Size-selection of the final SMRTbell library (if applicable)

Table 1 below shows the three main types of library supported in this document, by average insert size, with specifications for gDNA quality and quantity required for each.

Insert Size Target	Size Selection	gDNA Quality Required	Shearing Method	Range of Sheared and Concentrated Input gDNA (µg)	Recommended Sheared and Concentrated DNA Amount
~10 kb	No	Mode >20kb	g-TUBE	1.0 to 2.0 µg	1.0 µg
>15 kb (size-selected using BluePippin) system)	Yes	Mode > 40kb	g-TUBE or Megaruptor®	2.0 to 5.0 µg	3.0 µg
>30 kb (size-selected using BluePippin system)	Yes	Mode >50kb	26G needle or Megaruptor	3.0 to 5.0 µg	5.0 µg

Table 1: Recommended Shearing Methods and Input DNA for the Three Supported Library Types.

Depending on the quality of the sample, approximately 50% of the starting gDNA sample may be lost during the initial shearing and AMPure PB bead concentration steps. Therefore, we recommend starting this procedure with twice the sheared and concentrated gDNA amounts indicated in Table 1 above.

Part Number 101-693-800 Version 01 (January 2019)

Required Materials

Item	Where Required	Vendor	Part Number
**Options for High Molecular Weight DNA QC**			
CHEF Mapper XA	Option 1 for sample QC	Bio-Rad	170-3670
Pippin Pulse Power Supply	Option 2 for sample QC	Sage Science	PP10200
FEMTO *Pulse*	Option 3 for sample QC	Advanced Analytical Technologies	P-0003-0817
**Options for DNA Shearing**			
**g-TUBE shearing**	Suitable for 6 kb – 20 kb	Covaris	10145
**Megaruptor shearing**	Suitable for 10 kb – 60 kb	Diagenode	B06010001
Long Hydropores		Diagenode	E07010002
Hydrotubes		Diagenode	C30010018
**26G Blunt End Needle Shearing**	Suitable for >30 kb	SAI Infusion Technologies	B26-150
1 mL Luer-Lok Tip Syringe		Becton Dickinson	309628
**Library Construction**			
SMRTbell Express Template Prep Kit 2.0	For all genomic library construction	PacBio	100-938-900
Tube rotator (or equivalent)		VWR	10136-084
Qubit 3.0 Fluorometer (or equivalent)		Life Technologies	Q33216
Qubit dsDNA HS Assay Kit (or equivalent)		Life Technologies	Q32854
AMPure PB Beads		PacBio	100-265-900
Wide Orifice Tips<br>Tips LTS W-O 200UL Fltr RT-L200WFLR	Required for libraries >15 kb	Rainin	17014294
**Size Selection**			
BluePippin Size-Selection System	For size selection	Sage Science	BLU0001
BluePippin Gel Cassette and DNA Marker S1 Kit	For size-selection 10 kb – 30 kb	Sage Science	PAC20KB
BluePippin Gel Cassette and DNA Marker U1 Kit	For size-selection >30 kb	Sage Science	PAC30KB

Table 2. List of Required Materials and Equipment.

Overview of the SMRTbell Express 2.0 Large-insert Library Workflow

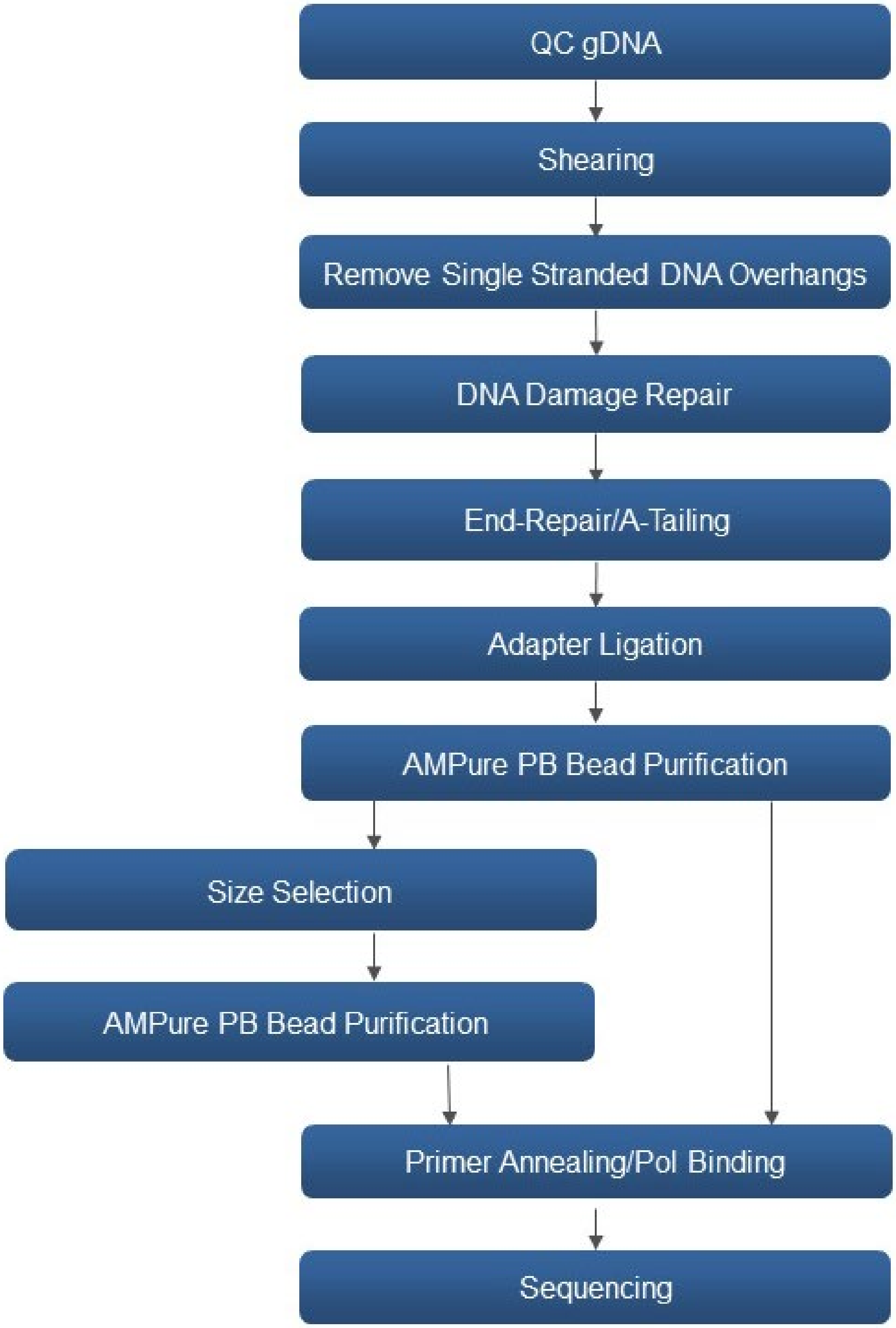

Figure 1: Workflow for Preparing Large-insert Libraries Using the SMRTbell Express Template Preparation Kit 2.0.

Best Practice Recommendations

1. For small numbers of samples, use DNA Lo-Bind 1.5 mL microcentrifuge tubes for all enzymatic and AMPure PB bead purification steps.
2. Multi-channel pipettes and PCR strip tubes may be used to efficiently process large numbers of samples.
3. Use wide-bore tips for all pipette mixing steps when preparing >15 kb and >30 kb size-selected libraries.
4. Never vortex tubes containing high molecular weight DNA samples. Mix by gentle pipetting or by flicking the tube.
5. Always follow best practices for DNA quantitation using a Qubit system:
   - Use the dsDNA high sensitivity reagent kit.
   - Prepare the Qubit working solution by diluting the Qubit reagent 1:200 in Qubit buffer. Prepare 200 µL of working solution for each standard and sample. Always prepare fresh standards for each assay.
   - Set up two 190 µL assay tubes for the standards and one 199 µL assay tube for each sample. Add 10 µL standard (from kit) and 1 µL sample to the respective assay tubes. Both the standard and sample DNAs should be at room temperature.
   - Vortex all tubes for 2 seconds.
   - Incubate the tubes for 2 minutes at room temperature prior to measurement.

Reagent Handling:

Several tubes in the kits (shown in Table 3 below) are sensitive to temperature and vortexing. We highly recommend:

- Never leaving tubes at room temperature.
- Working on ice at all times when preparing master mixes.
- Finger tapping followed by a quick-spin prior to use.

Reagent	Where Used
DNA Prep Additive	Remove single-strand overhangs
DNA Prep Enzyme	Remove single-strand overhangs
DNA Damage Repair Mix v2	DNA Damage Repair
End Prep Mix	End-Repair/A-tailing
Overhang Adapter v3	Ligation
Ligation Mix	Ligation
Ligation Additive	Ligation
Ligation Enhancer	Ligation

Table 3: Temperature sensitive reagents

Evaluate Genomic DNA (gDNA) Quality

To ensure success, gDNA size and integrity must be verified by pulsed field gel electrophoresis (PFGE) or an equivalent method before beginning library preparation. Any of the three commercially available systems listed in Table 4 below may be used to evaluate gDNA quality. Links to recommended procedures for each are also provided. Figure 2 shows typical results from gDNA quality assessment by Bio-Rad's CHEF Mapper pulsed field gel electrophoresis, which allows good resolution of high molecular weight gDNA.

Method	Procedure
Bio-Rad® CHEF Mapper® XA Pulsed Field Electrophoresis System	Procedure & Checklist - Using the BIO-RAD® CHEF Mapper® XA Pulsed Field Electrophoresis System
Advanced Analytical Technologies, Inc. FEMTO Pulse	Advanced Analytical Website
Sage Science Pippin Pulse	Procedure & Checklist - Using the Sage Science Pippin Pulse Electrophoresis Power Supply System

Table 4. gDNA Quality Evaluation Methods and Procedures.

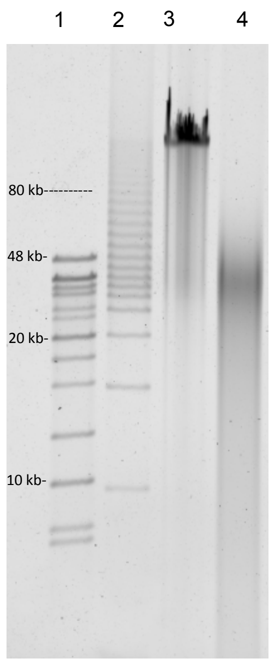

Lane 1: 8-48 kb Ladder (Bio-Rad)
Lane 2: 5 kb ladder (Bio-Rad)
Lane 3: HMW gDNA
Lane 4: Degraded gDNA

Figure 2: Evaluation of gDNA quality using Bio-Rad CHEF Mapper. Lane 3 is an example of a high quality, high molecular weight gDNA sample, where most of the DNA migrates as a single band at the top of the gel. Lane 4 shows an example of a partially degraded gDNA sample where most of the DNA migrates below ~50 kb and is thus not suitable for constructing a >30 kb size selected library. Depending on the application, the sample in Lane 4 (although fragmented) may be constructed to a SMRTbell library without shearing. If size selection is required, a less aggressive cutoff may be performed for this sample.

DNA Shearing

The SMRTbell library target size to construct depends on the goals of the project and the quality and quantity of the starting gDNA. For plant or animal whole genome assembly projects, it is highly recommended to start with high molecular weight gDNA (where majority of the DNA is >80 kb) which can be sheared and size-selected to 30 kb or larger.

In contrast, whole genome sequencing of smaller types of genomes (e.g., Class I/II microbes) may not necessarily need the same minimum library size requirements. For example, if the majority of the isolated gDNA fragments are >20 kb, construction of a >10 kb SMRTbell library may suffice to assemble a microbial-sized genome (if it does not contain highly complex repeats) to a single or a few contigs. See Procedure & Checklist – Preparing Multiplexed Microbial SMRTbell Libraries Using SMRTbell Express Template Prep Kit 2.0

When possible, Pacific Biosciences recommends performing test shears to determine the best shearing condition for your samples. The response of individual gDNA samples to recommended shearing parameters may differ and must be determined empirically and evaluated by PFGE or other suitable systems.

To ensure sufficient yields of final size-selected libraries, the input gDNA sample must be sheared so that the average size of the fragmented DNA remains well above the desired size-selection cut-off.

When preparing large insert SMRTbell libraries, the recommended yield and insert size distribution depends on the size selection lower cut-off to be employed. Table 5 below may be considered a useful starting point; but empirical optimization and accurate size quantitation are essential.

Nominal Target Library Insert Size	Size-Selection Lower Cut-off	Target DNA Shear Size
10 kb	6 kb (if required)	≥ 10 kb
20 kb	15 kb	≥ 20 kb
30 kb or 40 kb	20 kb	≥ 30 kb
50 kb	30 kb	≥ 50 kb
60 kb	40 kb	≥ 60 kb

Table 5. Size Selection Cut-off and Target Shearing Size Guidance for Constructing Large Insert Libraries.

Recommended methods for shearing gDNA to achieve a specified target fragment size are listed below:

Recommended Shearing Device	Target DNA Fragment Size	Page
g-TUBE (Covaris)	6 kb - 20 kb	6 - 7
Megaruptor (Diagenode)	10 kb - 75 kb	7 - 8
26G Needle	>30 kb	9 - 10

Table 6. Recommended Shearing Methods for Genomic DNA.

Covaris g-TUBE for Constructing 10 kb and 20 kb (>15 kb Size-Selected) SMRTbell Libraries

The g-TUBE can be used to shear gDNA to 6 kb - 20 kb fragments. Specific guidance (modified from the Covaris User Manual) for using g-TUBEs to construct 10 kb and 20 kb SMRT bell libraries are described below and should be strictly followed to achieve the desired target fragment size.

For 10 kb Libraries:
For constructing 10 kb libraries, we highly recommend following the validated shearing procedure below (which varies from the Covaris instructions) to shear gDNA to average 10 kb fragments.

Depending on the quality of the sample, approximately 50% of the starting gDNA sample may be lost during the initial shearing and AMPure PB bead concentration steps. Therefore, be sure to have sufficient amounts of starting gDNA in order to have at least 1 µg of sheared and concentrated DNA for the subsequent repair steps.

STEP	✓	Shear DNA	Notes
1		Dilute **2.0 µg** gDNA into **200 µL** 1X Elution Buffer to a final concentration of 10 ng/µL.	
2		Transfer gDNA to g-TUBE and centrifuge at 2400 x g (6000 rpm in the Eppendorf MiniSpin Plus) for 2 minutes.	
3		Repeat spin until entire gDNA sample has passed through the orifice. (This may take 2-3 spins).	
4		Invert the g-TUBE and centrifuge at 2400 x g (6000 rpm in the Eppendorf MiniSpin Plus) for 2 minutes.	
5		Repeat spin until entire gDNA sample has passed through the orifice. (This may take 2-3 spins).	
6		Transfer the sheared gDNA to a fresh 1.5 mL Lo-bind microfuge tube.	
7		Proceed to the “Concentrate DNA using AMPure PB Beads” section to concentrate the sample using AMPure PB beads.	

For 20 kb with >15 kb Size-Selected libraries:
To construct 20 kb libraries suitable for size selection (using a 15 kb lower cut-off with the BluePippin system), we highly recommend the following validated shearing procedure (which varies from the Covaris instructions).

Depending on the quality of the sample, approximately 50% of the starting gDNA sample may be lost during the initial shearing and AMPure PB bead concentration steps. Therefore, be sure to have sufficient amounts of starting DNA in order to have at least 3 µg of sheared and concentrated DNA for the subsequent repair steps.

STEP	✓	Shear DNA	Notes
1		Dilute gDNA to **200 ng/µL** - **300 ng/µL** in Elution Buffer. The sample volume may range from **20 µL** - **100 µL**.	
2		Transfer gDNA to g-TUBE and shear the gDNA at 2029 x g (5500 rpm in the Eppendorf MiniSpin Plus) for 2 minutes.	
3		Check for any residual sample remaining in the upper chamber. If present, re-spin for another 2 minutes. Repeat spin until entire gDNA sample has passed through the orifice.	
4		Invert and spin 2029 x g (5500 rpm in the Eppendorf MiniSpin Plus) until entire gDNA sample has passed through the orifice.	
5		Transfer the sheared gDNA to a fresh 1.5 mL Lo-bind microfuge tube.	
6		Proceed to the "Concentrate DNA using AMPure PB Beads" section to concentrate the sample using AMPure PB beads.	

Diagenode Megaruptor for Constructing >30 kb Size-Selected SMRTbell Libraries

PacBio highly recommends the Megaruptor system for shearing gDNA for constructing >30kb size selected libraries. Generally, we recommend following the manufacturer's recommendations for shearing gDNA using this method.

Depending on the quality of the sample, approximately 50% of the starting gDNA sample may be lost during the initial shearing and AMPure PB bead concentration steps. Therefore, be sure to have sufficient amounts of starting DNA in order to have at least 3 µg of sheared and concentrated DNA for the subsequent repair steps.

STEP	✓	Shear DNA	Notes
1		Dilute gDNA to **25 ng/µL** - **50 ng/µL** in Elution Buffer. The sample volume may range from **50 µL** - **400 µL**. It is important not to exceed this DNA concentration during shearing or the hydropore may become clogged. Before shearing, we recommend removing a 4 µL aliquot (un-sheared sample) for sizing QC.	
2		To shear gDNA for preparation of a >30 kb size-selected library, choose a target size of 50-60 kb in the Megaruptor software; for a >40 kb size-selected library, choose a target size of 75 kb. Use the Long Hydropores.	
3		Evaluate the size distribution of the resulting sheared gDNA fragments by running the un-sheared and sheared samples on a Bio-Rad® CHEF Mapper® XA Pulsed Field Electrophoresis system, AATI FEMTO Pulse or Sage Science Pippin Pulse.	
4		Proceed to the "Concentrate DNA using AMPure PB Beads" section to concentrate the sample using AMPure PB beads.	

Typical Megaruptor shearing results, for a high molecular weight bacterial gDNA sample, are shown in Figure 3. In this example, the 50kb shear setting (lane 4) was used to prepare a >30 kb size selected library, and the 75kb shear setting (lane 5) was used to prepare a >40 kb size-selected library.

If the gDNA sample appears under-sheared, try using a smaller target fragment size setting (for example, 40 kb for a >30 kb library) and/or a lower DNA concentration until you achieve a similar distribution of fragmented gDNA.

If the gDNA sample is over-sheared, try using a larger shear-size setting (for example, 75 kb for a >30 kb library).

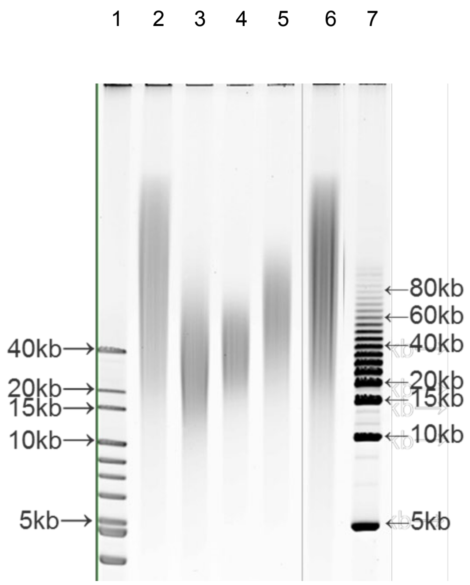

Lane 1: 1 kb extension DNA Ladder
Lane 2: Input K12 gDNA
Lane 3: g-TUBE sheared K12
Lane 4: 50 kb shear
Lane 5: 75 kb shear
Lane 6: Input K12 gDNA
Lane 7: Bio-Rad 5 kb DNA ladder

Figure 3: Evaluation of gDNA shears produced by Megaruptor. Both Lane 4 (50kb) and Lane 5 (75kb) are good distributions for constructing a large insert library with >30 kb and >40 kb size-selection, respectively. Sample in Lane 3 (g-TUBE) is over-sheared and not appropriate for >30 kb size-selection; in this case, using a 15 kb or 20 kb size-selection may be more appropriate.

Shearing using 26G Needles for Constructing >30 kb Size-Selected Libraries

Needle shearing is another option for constructing >30 kb size selected libraries. Before performing needle shearing, please view a short online video for demonstration on how to shear gDNA samples using 26G needles.

Depending on the quality of the sample, approximately 50% of the starting gDNA sample may be lost during the initial shearing and AMPure PB bead concentration steps. Therefore, be sure to have sufficient amounts of starting DNA in order to have at least 3 µg of sheared and concentrated DNA for the subsequent repair steps. The response of individual gDNA samples to the shearing parameters below may differ and must be determined empirically and evaluated by PFGE. Test shears are highly recommended.

Adjust the gDNA concentration to approximately 200-300ng/µL with Elution Buffer. If the initial DNA concentration is less than 200-300 ng/µL, concentrate the gDNA using AMPure PB beads prior to shearing.

STEP	✓	Shear DNA	Notes
1		Perform test shears by preparing a **50 µL** volume sample in a 1.5 mL LoBind tube. Remove a 1 µL aliquot (un-sheared sample) for use as a control when run on a pulsed-field gel electrophoresis gel for sizing QC.	
2		Aspirate the entire volume and pass the sample through a 26G needle five times, then remove a second 1 µL aliquot (5x sample) for sizing QC.	
3		Pass the sample through the needle five more times and remove a third 1 µL aliquot (10X sample) for sizing QC.	
4		Finally, pass the sample through the needle ten more times and remove a fourth 1 µL aliquot (20x sample) for sizing QC.	
5		Evaluate the size distribution of the resulting sheared gDNA fragments by running the un-sheared, 5X, 10X, and 20X samples on a Bio-Rad CHEF Mapper XA Pulsed Field Electrophoresis system, AATI FEMTO Pulse or Sage Science Pippin Pulse.	
6		Proceed to the "Concentrate DNA using AMPure PB beads" section to concentrate the sample using AMPure PB beads.	

Examples of gDNA sheared using the needle shearing method are shown in Figure 4. Samples in lane 5 (5X shears), lane 6 (10X shears) and lane 7 (20X shears) show good distribution above 40 kb that are suitable for size selection using 30 kb or 40 kb cut-offs.

If the gDNA sample appears under-sheared, decrease the DNA concentration (for example, try using 125 ng/µL) and/or increase the number of passes through the needle until you achieve a similar distribution of fragmented gDNA as shown in Lanes 5, 6 and 7.

If the gDNA sample is over-sheared, reduce the number of passes through the needle (e.g., try 1X and 2X).

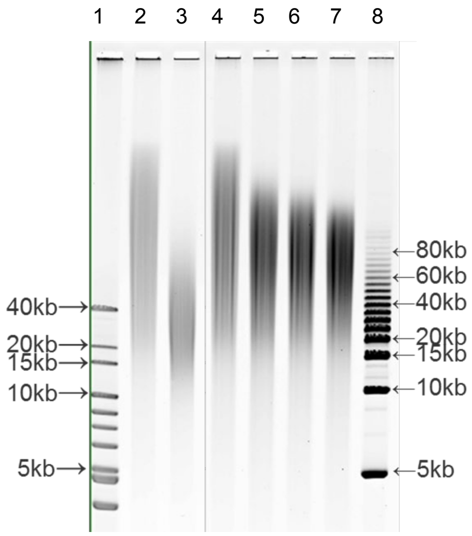

Lane 1: 1 kb Extension DNA Ladder
Lane 2: Input gDNA
Lane 3: g-TUBE sheared
Lane 4: Input gDNA
Lane 5: 26G_250ng/ul_5x shears
Lane 6: 26G_250ng/ul_10x shears
Lane 7: 26G_250ng/ul_20x shears
Lane 8: Bio-Rad 5 kb DNA ladder

Figure 4. Evaluation of gDNA shears produced by the needle shearing method. Samples in Lanes 5, 6, and 7 show good fragment distributions suitable for constructing a >30 kb or 40 kb size-selected library. The sample in Lane 3 was sheared with a g-TUBE device and is over-sheared and not appropriate for constructing a >30 kb size-selected library; using a 15 kb or 20 kb size-selection cutoff may be more appropriate.

Once the optimal shearing condition has been determined, scale up the shearing process by increasing the sample volume while also maintaining the same DNA concentration used during test shears.

Concentrate sheared gDNA using AMPure PB Beads

Bring all sheared gDNA samples to a minimum volume of 100 uL with 1X Elution Buffer, if necessary, before concentration. Wide bore pipette tips are required when constructing large insert libraries (>15 kb).

STEP	✓	Concentrate DNA	Notes
1		Add **0.45X** volume of AMPure PB magnetic beads to the sheared gDNA<br>_____µL of sample X **0.45X** =_______µL of beads<br>Note that the beads must be brought to room temperature before use and all AMPure PB bead purification steps should be performed at room temperature.<br>Before using, mix the bead reagent well until the solution appears homogenous. Pipette the reagent slowly since the bead mixture is viscous and precise volumes are critical to the purification process.	
2		Pipette mix 15 times with wide-bore pipette tips. It is important to mix well.	
3		Quickly spin down the tube (for 1 second) to collect the beads.	
4		Incubate the mix on bench top for 5 minutes at room temperature.	
5		Spin down the tube (for 1 second) to collect beads.	
6		Place the tube in a magnetic bead rack until the beads collect to the side of the tube and the solution appears clear. The actual time required to collect the beads to the side depends on the volume of beads added.	
7		With the tube still on the magnetic bead rack, slowly pipette off cleared supernatant and save in another tube. Avoid disturbing the beads.<br>If the DNA is not recovered at the end of this procedure, you can add equal volumes of AMPure PB beads to the saved supernatant and repeat the AMPure PB bead purification steps to recover the DNA.	
8		Wash beads with freshly prepared 80% ethanol.<br>Note that 80% ethanol is hygroscopic and should be prepared FRESH to achieve optimal results. Also, 80% ethanol should be stored in a tightly capped polypropylene tube for no more than 3 days.<br>– Do not remove the tube from the magnetic rack.<br>– Use a sufficient volume of 80% ethanol to fill the tube (1.5 mL for 1.5 mL tube or 2 mL for 2 mL tube). Slowly dispense the 80% ethanol against the side of the tube opposite the beads.<br>– Do not disturb the beads.<br>– After 30 seconds, pipette and discard the 80% ethanol.	
9		Repeat step 8.	
10		Remove residual 80% ethanol.<br>– Remove tube from magnetic bead rack and spin. Both the beads and any residual 80% ethanol will be at the bottom of the tube.<br>– Place the tube back on magnetic bead rack and allow the beads to separate.<br>– Pipette off any remaining 80% ethanol.	
11		Check for any remaining droplets in the tube. If droplets are present, repeat step 10.	

STEP		Concentrate DNA	Notes
**12**		The volume to use for elution is **46 µL** (the volume to go into “Remove Single-Stranded DNA Overhangs”).	
**13**		Add the Elution Buffer volume to your beads. Pipette mix 15 times with wide-bore pipette tips. It is important to mix well.<br>– Place at 37°C for 15 minutes to elute the DNA from the beads.<br>– Spin the tube down, then place the tube back on the magnetic bead rack.<br>– Let beads separate fully. Then without disturbing the beads, transfer supernatant to a new 1.5 ml Lo-Bind tube.<br>– Discard the beads.	
**14**		Verify your DNA amount and concentration using a Qubit quantitation platform.<br>– Measure the DNA concentration using a Qubit fluorometer.<br>– Using 1 µL of the eluted sample, make a 1:10 dilution in EB.<br>– Use 1 µL of this 1:10 dilution to measure the DNA concentration using the dsDNA HS Assay kit according to the manufacturer’s recommendations.<br>The remaining 9 µL of 1:10 diluted sample may be used for QC by pulsed field gel electrophoresis.	
**15**		The sheared DNA can be stored for up to 2 weeks at 4°C or at -20°C for longer duration. Do not freeze/thaw.	
**16**		Actual recovery per µL and total available sample material: ____	

Remove Single-Strand Overhangs

Before starting with the procedure, please refer to Table 3 for reagents handling. Always work in ice.

Use the following table to set up a reaction to remove single-strand overhangs using up to 5 µg of input sheared gDNA. If starting with more than 5 µg of sheared gDNA, scale the reaction volumes proportionally (i.e., for a mass between 5-10 µg of DNA, scale the total volume to 110 µL).

Wide bore pipette tips are required when constructing large insert libraries (>15 kb).

The table below shows that the minimum required amount of input sheared gDNA varies depending on the target SMRTbell library insert size:

Library Size	Recommended Input Sheared gDNA* (µg)	Minimum Input Sheared gDNA* (µg)	Maximum Input Sheared gDNA* (µg)
10 kb library - no size selection or with size selection	1.0	1.0	5.0
>15 kb library with size selection	3.0	2.0	5.0
>30 kb library with size selection	5.0	3.0	5.0

* Input DNA amount determined by Qubit dsDNA HS assay.

Table 7. Minimum and Maximum Input Sheared gDNA into the first enzymatic reaction.

Follow the steps below for removal of single-strand overhangs:

1. The stock DNA Prep Additive must first be diluted. Dilute the stock DNA Prep Additive 1:5 in Enzyme Dilution Buffer (found in the kit). Dilute 1 µL of stock DNA Prep Additive into 4 µL Enzyme Dilution Buffer. The diluted DNA Prep Additive should be used immediately and should not be stored.
2. Prepare the following reaction.

Reagent (Reaction Mix 1)	Tube Cap Color	Volume	✓	Notes
DNA Prep Buffer	(yellow)	7.0 µL		
Sheared DNA		45.0 µL		
NAD	(red)	1.0 µL		
Diluted DNA Prep Additive (see step 1)	(purple)	1.0 µL		
DNA Prep Enzyme	(blue)	1.0 µL		
Total Volume		55.0 µL		

3. Pipette mix 10 times with wide-bore pipette tips. It is important to mix well.
4. Spin down contents of tube with a quick spin in a microfuge.
5. Incubate at 37°C for 15 minutes, then return the reaction to 4°C. Proceed to the next step.

DNA Damage Repair

Use the following table to prepare your reaction.

Reagent (Reaction Mix 2)	Tube Cap Color	Volume	✓	Notes
Reaction Mix 1		55.0 µL		
DNA Damage Repair Mix v2		2.0 µL		
Total Volume		57.0 µL		

1. Pipette mix 10 times with wide-bore pipette tips. It is important to mix well.
2. Spin down contents of tube with a quick spin in a microfuge.
3. Incubate at 37°C for 30 minutes, then return the reaction to 4°C. Proceed to the next step.

End-Repair/A-tailing

Use the following table to prepare your reaction.

Reagent (Reaction Mix 3)		Volume	✓	Notes
Reaction Mix 2		57.0 µL		
End Prep Mix		3.0 µL		
Total Volume		60.0 µL		

1. Pipette mix 10 times with wide-bore pipette tips. It is important to mix well.
2. Spin down contents of tube with a quick spin in a microfuge.
3. Incubate at 20°C for 10 minutes.
4. Incubate at 65°C for 30 minutes, then return the reaction to 4°C.
   Proceed to the next step.

Adapter Ligation

Use the following table to prepare your reaction, adding the components below in the order listed.

Reagent (Reaction Mix 4)	Tube Cap Color	Volume	✓	Notes
Reaction Mix 3		60.0 µL		
Overhang Adapter v3		5.0 µL		
Ligation Mix		30.0 µL		
Ligation Additive		1.0 µL		
Ligation Enhancer		1.0 µL		
Total Volume		97.0 µL		

1. Pipette mix 10 times with wide-bore pipette tips. It is important to mix well.

2. Spin down contents of tube with a quick spin in a microfuge.
3. Incubate at 20°C for 60 minutes, then return the reaction to 4°C. Proceed to the next step.

Purify SMRTbell Templates

STEP	✓	Purify DNA	Notes
1		Add **44 µL** (**0.45X**) volume of AMPure PB beads to the **97 µL** Reaction Mix 4**.**	
2		Mix the bead/DNA solution thoroughly by pipette mixing 15 times with wide-bore pipette tips. It is important to mix well.	
3		Quickly spin down the tube (for 1 second) to collect the beads.	
4		Incubate samples on bench top for 5 minutes at room temperature.	
5		Spin down the tube (for 1 second) to collect beads.	
6		Place the tube in a magnetic bead rack to collect the beads to the side of the tube.	
7		Slowly pipette off cleared supernatant and save (in another tube). Avoid disturbing the beads.	
8		Wash beads with freshly prepared 80% ethanol.<br><br>Note that 80% ethanol is hygroscopic and should be prepared FRESH to achieve optimal results. Also, 80% ethanol should be stored in a tightly capped polypropylene tube for no more than 3 days.<br><br>– Do not remove the tube from the magnetic rack.<br>– Use a sufficient volume of 80% ethanol to fill the tube (1.5 mL for a 1.5 mL DNA LoBind tube)<br>– Slowly dispense the 80% ethanol against the side of the tube opposite the beads.<br>– Do not disturb the beads.<br>– After 30 seconds, pipette and discard the 80% ethanol.	
9		Repeat **step 8**.	
10		Remove residual 80% ethanol.<br><br>– Remove tube from magnetic bead rack and spin. Both the beads and any residual 80% ethanol will be at the bottom of the tube.<br>– Place the tube back on magnetic bead rack and allow beads to separate.<br>– Pipette off any remaining 80% ethanol.	
11		Check for any remaining droplets in the tube. If droplets are present, repeat step 10.	
12		Immediately add **the appropriate volume** (see below) of Elution Buffer volume to the beads to elute the DNA.<br><br>(see table below)<br><br>– Pipette mix 15 times with wide-bore pipette tips. It is important to mix well.<br>– Elute the DNA by letting the mix incubate at 37 ºC for 15 minutes. This is important to maximize recovery of high molecular weight DNA.<br>– Spin the tube down, then place the tube back on the magnetic bead rack.<br>– Let beads separate fully. Then, without disturbing the beads, transfer the supernatant to a new 1.5 ml Lo-Bind tube.<br>– Discard the beads.	

	**Elution Volume**
Libraries requiring size-selection	31 µL
Libraries requiring no size-selection	11 µL

**13**		Verify your DNA amount and concentration using a Qubit quantitation platform.<br>– Measure the DNA concentration using a Qubit fluorometer.<br>– Using 1 µL of the eluted sample, make a 1:10 dilution in EB.<br>– Use 1 µL of this 1:10 dilution to measure the DNA concentration using a Qubit fluorometer and the dsDNA HS Assay kit according to the manufacturer's recommendations.	
**14**		Actual recovered DNA SMRTbell concentration (ng/µL): ___________________<br>Total recovered DNA SMRTbell amount (ng): ______________________	
**15**		**For samples that do not require size selection,** the library is ready for primer annealing and polymerase binding. Proceed to the 'Anneal and Bind SMRTbell Library Templates' section of this procedure. Unannealed library material can be stored for up to 2 weeks at 4°C or at -20°C for longer duration. Do not freeze/thaw.<br><br>**For samples requiring size selection**, it is highly recommended to perform qualitative and quantitative analysis using Pulse Field Gel Electrophoresis before size selection. This step will allow you to choose the appropriate Blue Pippin cut-off for size selection. Choosing an aggressive BP cutoff prior to determining size distribution of SMRTbell Templates might lead to significant sample loss.	
**16**		**For samples requiring size selection**, proceed with size selection using the BluePippin system. If the library cannot undergo size selection immediately, it can be stored for up to 2 weeks at 4°C or at -20°C for longer duration. Do not freeze/thaw.	

Size Selection with the BluePippin System

When constructing large insert SMRTbell libraries for whole genome sequencing of complex organisms, it is beneficial to remove small insert SMRTbell templates by performing size selection with the BluePippin System (which collects fragments above a size cut-off threshold). With the BluePippin system, the BP Start (cut-off) value may be adjusted depending on the size distribution and total available mass of the SMRTbell library. The table below shows recommended Blue Pippin run setup protocols for size selection. For the latest BluePippin User Manual and guidance on size-selection protocols, please contact Sage Science (www.sagescience.com).

Target lower cut-off threshold (BP Start)	Cassette Definition File	Min. Input DNA per Lane	Marker	BP End (bp)	Run Time
6 - 10 kb	0.75% DF Marker S1 high-pass 6-10 kb vs3	>500 ng	S1	50000	3-4 hrs
15 - 20 kb	0.75% DF Marker S1 high-pass 15 kb -20kb	>2.0 µg	S1	50000	5.5 hrs
30 - 40 kb	0.75% DF Marker U1 high-pass 30-40kb vs3	>2.0 µg	U1	80000	10.0 hrs

Table 8. Recommended Blue Pippin Run Setup Protocols for Size Selection.

Note: Visit Sage's website (http://www.sagescience.com) to verify that your BluePippin software is up-to-date. The current version is v6.31

<table>
<tr><th>STEP</th><th>✓</th><th>BluePippin Size Selection</th><th>Notes</th></tr>
<tr><td>1</td><td></td><td>Prepare up to 5 µg SMRTbell templates in a final volume of 30 µL Elution Buffer for each Blue Pippin lane.</td><td></td></tr>
<tr><td>2</td><td></td><td>Bring the Loading Solution to room temperature, and then add 10 µL of the Loading Solution to the 30 µL DNA sample. For loading multiple lanes with the same sample, scale the volumes proportionally. The Loading Solution is viscous, so pipet slowly to ensure complete transfer into the DNA sample.
a. Pipette mix using wide-bore pipette tips to mix.
b. Spin briefly to collect the contents at the bottom of the tube.</td><td></td></tr>
<tr><td>3</td><td></td><td>Follow the manufacturer’s recommendations to set up a run protocol.
a. When setting up the run protocol, select the appropriate Cassette Definition File for your sample.
b. Using the “Range” selection mode, enter the desired “BPstart” value. A “BP End” value should automatically appear.
Note: *When using the ‘0.75% DF Marker S1 high-pass 15 kb -20kb’ and ‘0.75% DF Marker U1 high-pass 30-40kb vs3’ cassette definition file, sample lanes containing <3 µg of SMRTbell library material will run faster during electrophoresis. In such cases, PacBio recommends adjusting the BP Start values as follow:
<table>
<tr><th>Cassette Definition File</th><th>If < 3 ug input per lane, use BP Start</th></tr>
<tr><td rowspan="2">0.75% DF Marker S1 high-pass 15 kb - 20kb</td><td>12500 for 15 kb cutoff</td></tr>
<tr><td>15000 for 20 kb cutoff</td></tr>
<tr><td rowspan="2">0.75% DF Marker U1 high-pass 30 -40kb vs3</td><td>25000 for 30 kb cutoff</td></tr>
<tr><td>35000 for 40 kb cutoff</td></tr>
</table>
c. Be sure to assign a marker lane. We recommend using Lane 4 for the marker.</td><td></td></tr>
<tr><td>4</td><td></td><td>Load the samples into the BluePippin gel cassette in Lanes 1, 2, 3, or 5. PacBio recommends loading the appropriate DNA Marker in Lane 4.</td><td></td></tr>
<tr><td>5</td><td></td><td>To maximize recovery of eluted DNA, wait at least 30 minutes after the run terminates before removing the sample from the elution chamber.
a. Collect the eluate into a 1.5 mL DNA LoBind tube.
b. Wash the elution well with 40 µL of Sage Science’s 0.1% Tween-20 Wash Solution and then add the recovered wash liquid to the eluted sample. Washing the elution well may further increase recovery yields by approximately 10-20%.</td><td></td></tr>
</table>

Purification of SMRTbell Templates after Size Selection

Bring up the volume of eluted, size-selected DNA SMRTbell templates to 100 µL with 1X Elution Buffer. Wide bore pipette tips are required when constructing large insert libraries (>15 kb).

STEP	✓	Purify DNA	Notes
1		Add **100 µL** (**1X**) volume of AMPure PB beads to the **100 µL** solution of size-selected DNA SMRTbell library.	
2		Mix the bead/DNA solution thoroughly by pipette mixing 15 times with wide-bore pipette tips. It is important to mix well.	
3		Quickly spin down the tube (for 1 second) to collect the beads.	
4		Incubate samples on bench top for 5 minutes at room temperature.	
5		Spin down the tube (for 1 second) to collect beads.	
6		Place the tube in a magnetic bead rack to collect the beads to the side of the tube.	
7		Slowly pipette off cleared supernatant and save (in another tube). Avoid disturbing the beads.	
8		Wash beads with freshly prepared 80% ethanol.<br>Note that 80% ethanol is hygroscopic and should be prepared FRESH to achieve optimal results. Also, 80% ethanol should be stored in a tightly capped polypropylene tube for no more than 3 days.<br>– Do not remove the tube from the magnetic rack.<br>– Use a sufficient volume of 80% ethanol to fill the tube (1.5 mL for a 1.5 mL DNA LoBind tube)<br>– Slowly dispense the 80% ethanol against the side of the tube opposite the beads.<br>– Do not disturb the beads.<br>– After 30 seconds, pipette and discard the 80% ethanol.	
9		Repeat **step 8**.	
10		Remove residual 80% ethanol.<br>– Remove tube from magnetic bead rack and spin. Both the beads and any residual 80% ethanol will be at the bottom of the tube.<br>– Place the tube back on magnetic bead rack and allow beads to separate.<br>– Pipette off any remaining 80% ethanol.	
11		Check for any remaining droplets in the tube. If droplets are present, repeat step 10.	
12		Immediately add **11 µL** of Elution Buffer volume to your beads. Pipette mix 15 times with wide-bore pipette tips. It is important to mix well.<br>– Elute the DNA by letting the mix incubate at 37 ºC for 15 minutes. This is important to maximize recovery of high molecular weight DNA.<br>– Spin the tube down, then place the tube back on the magnetic bead rack.<br>– Let beads separate fully. Then without disturbing the beads, transfer supernatant to a new 1.5 ml Lo-Bind tube.<br>– Discard the beads.	
13		Verify your DNA amount and concentration using a Qubit quantitation platform.<br>– Measure the DNA concentration using a Qubit fluorometer.<br>– Using 1 µL of the eluted sample, make a 1:10 dilution in EB.<br>– Use 1 µL of this 1:10 dilution to measure the DNA concentration using a Qubit fluorometer and the dsDNA HS Assay kit according to the manufacturer's recommendations.	
14		Actual recovered DNA SMRTbell concentration (ng/µl): ____________________<br>Total recovered DNA SMRTbell amount (ng): _______________________	

Anneal and Bind SMRTbell Library Templates

Use SMRT Link Sample Setup for instructions for primer annealing and polymerase binding.
For primer annealing, use **Sequencing Primer v4** (found in the SMRTbell Express Template Prep Kit v2).
Sequencing Primer v4 is for diffusion loading only and cannot be used for MagBead loading.

Prepare for Sequencing

For the Sequel System, Diffusion loading is recommended for loading large insert libraries prepared by the SMRTbell Express Template Prep Kit v2.
Follow the Sample Setup in SMRT Link for preparing your sample for sequencing.

For detailed recommendations for sequencing of specific library insert size ranges, refer to the Quick Reference Card – Diffusion Loading and Pre-Extension Time Recommendations for the Sequel System here.

Revision History (Description)	Version	Date
Initial Release.	01	January 2019

For Research Use Only. Not for use in diagnostic procedures. © Copyright 2019, Pacific Biosciences of California, Inc. All rights reserved. Information in this document is subject to change without notice. Pacific Biosciences assumes no responsibility for any errors or omissions in this document. Certain notices, terms, conditions and/or use restrictions may pertain to your use of Pacific Biosciences products and/or third party products. Please refer to the applicable Pacific Biosciences Terms and Conditions of Sale and to the applicable license terms at http://www.pacificbiosciences.com/licenses.html. Pacific Biosciences, the Pacific Biosciences logo, PacBio, SMRT, SMRTbell, Iso-Seq and Sequel are trademarks of Pacific Biosciences. BluePippin and SageELF are trademarks of Sage Science, Inc. NGS-go and NGSengine are trademarks of GenDx. FEMTO Pulse and Fragment Analyzer are trademarks of Advanced Analytical Technologies. All other trademarks are the sole property of their respective owners.
