# Supplementary material for: Case Report: A Novel Gross Deletion in PAX3 (10.26 kb) Identified in a Chinese Family With Waardenburg Syndrome by Third-Generation Sequencing
Source: Front Genet. 2021 Aug 11;12:705973. doi: 10.3389/fgene.2021.705973 (PMC8385755; doi:10.3389/fgene.2021.705973)
Supplement: Supplementary file 2 [file Table_1.DOCX]

**Supplementary Table 1**. Primer pairs of *PAX3*.

| **Primer** | **Sequences (5’→3’)** | **Primer** | **Sequences (5’→3’)** |
| --- | --- | --- | --- |
| PAX3-ex1 f | TCTGGACTAGGAACCGACAG | PAX3-ex1 r | TTCCTGGAAGCACCAAAGG |
| PAX3-ex2 f | GGTGCTGTAACATGAGGATAGG | PAX3-ex2 r | GGCAAGAGCTTTCCCTAAGT |
| PAX3-ex3 f | GCCCGCCTGTTCTCTTAAA | PAX3-ex3 r | AGAGGCCACCTCCCAATA |
| PAX3-ex4 f | AAGGATGAGGATGTCCAGAGA | PAX3-ex4 r | GCCGTCAGATCACCAATGT |
| PAX3-ex5 f | TGCACTGTAGACCAGGATTTG | PAX3-ex5 r | GAAGTAGGACACGGAGGTTTG |
| PAX3-ex6 f | GGCAGAGAACTCTTGCATCTAT | PAX3-ex6 r | CAGAGAAATCGCCTGGAAGTTA |
| PAX3-ex7 f | AGGTGTGACTGTATCTGTTATGG | PAX3-ex7 r | AGTAGGAAGGGTGGAGAGAA |
| PAX3-ex8 f | GTCTCCTGGACAGCTCTTTAAC | PAX3-ex8 r | GTGTGGCTTAATCTTGCCTCTA |
| PAX3-ex9 f | AGCCTTTGATAGCACGGTATT | PAX3-ex9 r | CTTCCTCTTCTCCACTGCTTT |
| primer 1 f | CAAGAAGAGGAGCCAGGTTT | primer 1 r | CAGAGCAGCCAGTGAGATAAG |
| primer 2 f | CCTGCTTGTCTCAACCATGT | primer 2 r | CCGTCAGATCACCAATGTCAG |
