# Supplementary material for: Case Report: A Novel Gross Deletion in PAX3 (10.26 kb) Identified in a Chinese Family With Waardenburg Syndrome by Third-Generation Sequencing
Source: Front Genet. 2021 Aug 11;12:705973. doi: 10.3389/fgene.2021.705973 (PMC8385755; doi:10.3389/fgene.2021.705973)
Supplement: Supplementary file 3 [file Table_2.DOCX]

**Supplementary Table 2**. CNVs were detected in I:1 by SNP-array.

| **CNV** | **Position** | **Size** | **Value** | **Gene** |
| --- | --- | --- | --- | --- |
| 1p36.21 duplication | chr1:12897312-12924881 | 26.9 kb | 3 | *LOC649330*, *HNRNPCL1*, *PRAMEF2* |
| 6p21.32 deletion | chr6:33024499-33037060 | 12.3 kb | 1 | *HLA-DPA1* |
| 6q26 deletion | chr6:161269903-161283051 | 12.8 kb | 1 | No |
| 8q24.3 deletion | chr8:144911463-144948861 | 36.5 kb | 1 | *PUF60*, *NRBP2*, *EPPK1* |
| 9q34.3 deletion | chr9:139916016-139960782 | 43.7 kb | 1 | *ABCA2*, *C9ORF139*, *FUT7*, *NPDC1*, *ENTPD2*, *SAPCD2* |
| 9q34.3 deletion | chr9:140087121-140123302 | 35.3 kb | 1 | *TPRN*, *TMEM203*, *NDOR1*, *RNF208*, *AK128153*, *C9ORF169*, *RNF224* |
| 11q13.1 deletion | chr11:65272220-65368290 | 93.8 kb | 1 | *MALAT1*, *MIR548AR*, *SCYL1*, *LTBP3*, *U7*, *SSSCA1-AS1*, *SSSCA1*, *FAM89B*, *EHBP1L1*, *KCNK7*, *MAP3K11* |

CNV, copy number variation; No, No gene in this locus.
