# Supplementary material for: Case Report: A Novel Gross Deletion in PAX3 (10.26 kb) Identified in a Chinese Family With Waardenburg Syndrome by Third-Generation Sequencing
Source: Front Genet. 2021 Aug 11;12:705973. doi: 10.3389/fgene.2021.705973 (PMC8385755; doi:10.3389/fgene.2021.705973)
Supplement: Supplementary file 4 [file Table_3.DOCX]

**Supplementary Figure legends**

**Fig. S1** The Visualization of whole-exome sequencing result of Chr2: 223152000-22317000 in the family by IGV software. Red words represent the affected members; the red box highlights the region with low coverage in patients.

**Fig. S2** Circus of structural variants distributing in chromosomes, detected by third generation sequencing. From the outside in, the first ring represents chromosome numbers and positions, the second, third, fourth and fifth circles successively show deletions, insertions, inversions and duplications, and the inner ring exhibited translocations.
