# Supplementary figures and images for: Case Report: A Novel Gross Deletion in PAX3 (10.26 kb) Identified in a Chinese Family With Waardenburg Syndrome by Third-Generation Sequencing
Source: Front Genet. 2021 Aug 11;12:705973. doi: 10.3389/fgene.2021.705973 (PMC8385755; doi:10.3389/fgene.2021.705973)

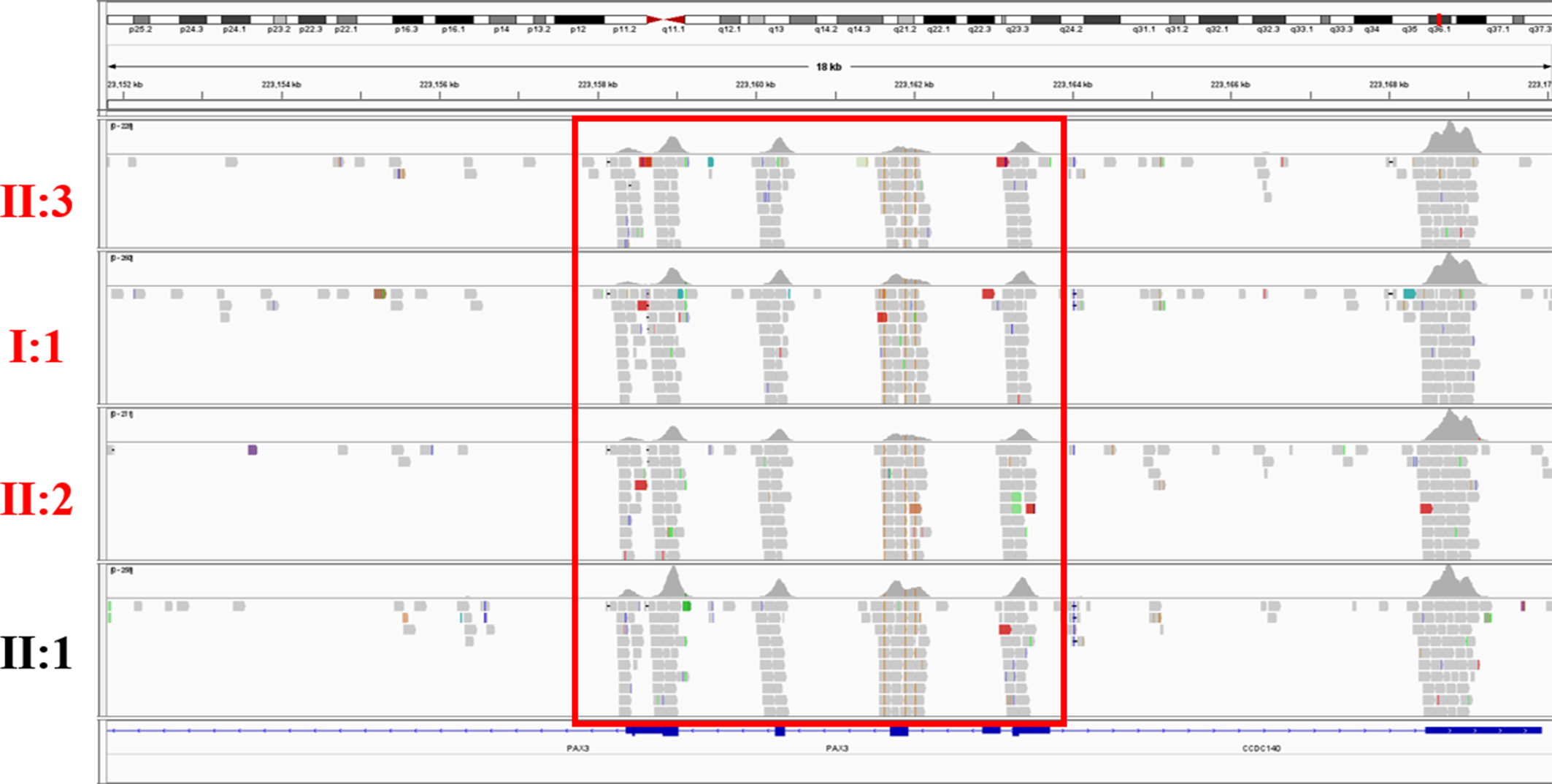

Supplement: Supplementary file 5 [file Image_1.TIF]

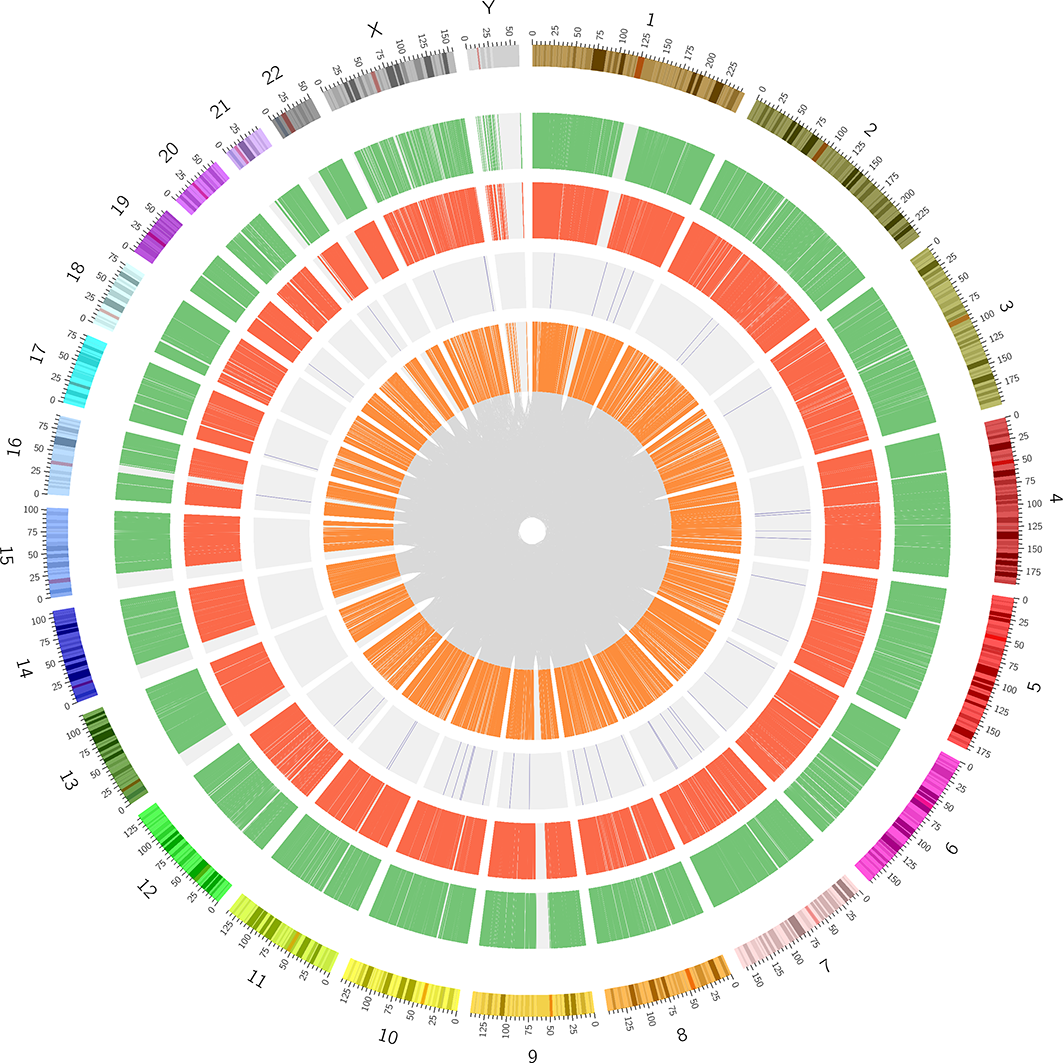

Supplement: Supplementary file 6 [file Image_2.TIF]
